# Kinematics and joints moments profile during straight arm press to handstand in male gymnasts

**DOI:** 10.1371/journal.pone.0253951

**Published:** 2021-07-14

**Authors:** Hisashi Mizutori, Yu Kashiwagi, Noriko Hakamada, Yasunori Tachibana, Kazuo Funato

**Affiliations:** 1 Japan Gymnastics Association, Shinjuku, Japan; 2 Senshu University Institute of Sport, Kawasaki, Kanagawa, Japan; 3 Japan Institute of Sports Sciences, Itabashi, Tokyo, Japan; 4 Japan Gymnastics Association, Shinjuku, Japan; 5 Graduate School of Sport System, Kokushikan University, Setagaya, Tokyo, Japan; Toronto Rehabilitation Institute - UHN, CANADA

## Abstract

Biomechanical features of the handstand, one of the most fundamental skills required for artistic gymnastics events, have not been well documented. The purpose of this study was to clarify the kinematics and joint moment profiles during straight arm press to handstand in different highly skilled male gymnasts. Fifty-nine male gymnasts performed a straight arm press to handstand on a force platform and were judged on their performance by experienced certified judges. Subjects were divided into two groups (highly-skilled and less-skilled). Kinematic data were obtained using a video camera synchronized with force platform. Joint moments (wrist, shoulder, hip) during each straight arm press to handstand were calculated using the inverse dynamics solution. Larger shoulder flexion moments were observed in less-skilled compared with highly- skilled performers (at 3–59%, *p* < 0.001) while larger hip flexion moments were observed in highly- skilled performers at 52% (*p* = 0.045) and 56% (*p* = 0.048) and normalized time of straight arm press to handstand. Major differences between highly-skilled and less-skilled performers were observed in hip joint moment production as it shifted from extension to flexion from the leg horizontal position to the handstand position in highly-skilled gymnasts. Successful straight arm press to handstand techniques observed in highly-skilled performers were characterized as a more acute pike position at toe-off as well as hip flexor moments at latter phase of the straight arm press to handstand.

## Introduction

**Fig 1:**
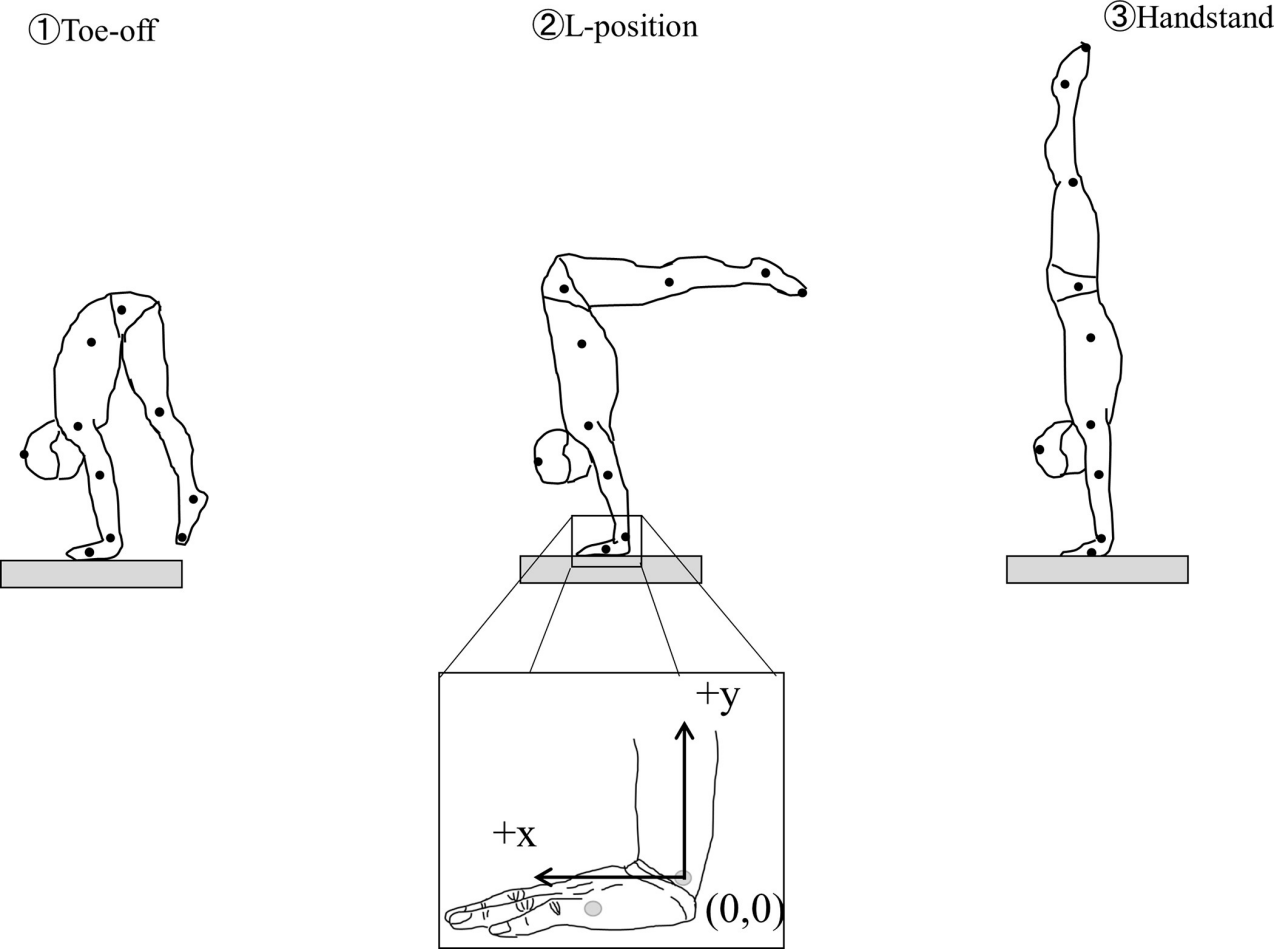
Typical straight arm press to handstand movement and three timings. ① Toe-off, ② Leg horizontal position, ③ Handstand. Origin of coordinate system was set as styloid process of ulna.

Piked body, straight arm press to handstand with legs together, extended at the hip from toe-off in a bent body position (straight arm press to handstand; SAPH), is a required skill for all apparatus except in the vault in Men’s artistic gymnastics [1]. It involves bending forward from a standing position with elbows extended, placing both hands on the floor, and, in this position, forming a handstand without recoiling (Fig 1). In Men’s artistic gymnastics competitions, SAPH has been incorporated as a standard movement in numerous routines on the floor, rings, parallel bars to the pommel horse dismount and the horizontal bar “Endo” maneuver [2]. Until 2005, as scoring rules limited the highest score to ten points [3] and, depending on the capabilities of the individual, gymnasts were permitted to omit the SAPH from their routines. However, scoring rules utilizing “D-scores”, evaluating the difficulty of an element and “E-scores”, were incorporated beginning in 2006 [4]. Due to the current rules [2], it became difficult to be award a high score without the gymnasts carrying out numerous very difficult elements. SAPH is one of the necessary factors included in a routine requisite to being awarded a high score.

The SAPH can be regarded as a “Strength Skill” in accordance with a floor, rings and parallel bars training classification [2]. Additionally, since the SAPH uses neither swing nor recoil, it is an element reliant upon the gymnast’s adjustment and production of muscle strength. Under the current rules [2], deduction scoring system regarding handstand skills defined as “Execution deductions for hesitation, strength, bent arms, lowering of the hips, and unsteadiness must be considered during the rise to the full handstand”. Therefore, the SAPH is evaluated as a good element when performed as an exercise of good balance rather than one of muscle strength. Rohleder, J. and Vogt, T. [5–7] have examined the learning of skill acquisition related to handstand task in gymnastics and given some useful empirical evidences based on coaches’ experiences in gymnastics. According to coaches involved in training for the handstand [8], skilled athletes put their center of mass (COM) just below their back and shoulders, performing the element beautifully without the use of excessive muscle strength.

Focus on studies of handstand biomechanics has been related to whether handstand balance adjustment strategies, employed when the body is inverse, are similar to those used when standing upright [9–13]. The magnitude of the moment produced by each joint during a handstand is from 0.2 Nm・kg^-1^ of body weight to 1.0 Nm・kg^-1^ at most [9]. However, during the SAPH, shoulder flexion as well as hip extension from toe-off to a straight body position is a more dynamic maneuver. As for joint moment analysis on SAPH during the parallel bars event [14–16], the shoulder joint moment is about three times body weight and hip and shoulder moments were discussed in terms of its importance in the SAPH balance strategies [9–12]. Furthermore, Prassas [14–16] discussed the importance of flexibility in the hip joint’s range of motion (ROM) during SAPH, suggesting that hip joint flexion is related to regulating the shoulder joint moment. Despite being an element necessary for raising the difficulty score of artistic gymnastics routines, there is currently a lack of scientific information on the SAPH. From a training [17–20] and a research perspective [8, 14–17, 21, 22] position of the shoulders and movement of the hip are very important aspects of the SAPH execution.

The goal of this project was to investigate whether skill level differences in the SAPH, among gymnastics, can be identified by joint moment patterns at selected individual joints. This could have been achieved by evaluating wrist, shoulder, and hip joint moments focusing on hip extension moment during the floor SAPH. Considering different skill levels, a measurable difference in moment magnitude and timing for each joint was expected [9].

According to Faria [17], the ability to raise the buttocks while maintaining a large flexor moment at the shoulder is a skill required to the successful performance of the SAPH. One might predict that highly skilled athletes will perform the movement focusing on moment patterns at the hip joint while maintaining a fully flexed position at the hip. On the other hand, less-skilled athletes, being unable to maintain full flexion, will have their shoulders in a forward position to preserve balance. Our hypothesis is that the magnitude of the shoulder joint moment will be greater in less-skilled athletes when compared to highly skilled athletes. The purpose of this study was to analyze the characteristics of kinematic and joint moment profiles during SAPH in different skill levels in a group of Japanese top class and junior level male gymnasts.

## Methods

### Participants

Fifty-nine male gymnastics (Age:16.4 ± 2.9years, Body Height (BH): 155.1 ± 12.5cm, Body Weight (BW): 50.6 ± 12.1kg) participated in the study, including forty-eight junior elite national gymnasts in national training camp. Subjects were selected according to following criterions: within sixth position of gymnastics individual synthesis at Japan senior high school gymnastic competitions and continued to perform the practice in several elite colleges in Japan. After explaining the purpose and contents of this study to participants their informed consent was obtained. In the case of minors, aims and content of the study were explained to parental guardians and team coaches before the consent from was obtained verbally. This research was carried out with approval from the Nippon Sport Science University’s ethical review board (approval number: 013-H52). Prior to beginning the experiments, trials of SAPH for each participant were observed and graded for judging qualifications by five judges, each with a minimum of ten years of competitive artistic gymnastics experience. The study was conducted in a special gymnasium for official gymnastics competitions. Official licensed five judges were positioned and judged as like in official competitions. The SAPHs were graded using scores ranging from 1(poor) to 5(excellent). Participants were divided into two groups; with those average scoring 4 and over were classified as the highly-skilled group (n = 29; Age:16.4 ± 2.9years, BH:154.2. ± 12.5cm, BW:49.9 ± 12.1kg) and those average scoring less than 4 as the less-skilled group (n = 30; Age:16.7 ± 2.1years, BH:156.4 ± 9.6cm, BW:51.8 ± 10.7kg). Statistically significant differences were not observed when considering the physical characteristics of both groups. Considering Type I error caused in statistical process, sample size was calculated by G*power software 3.1 set as significance (α) = 0.05, effect size (d) = 0.8 and power (1-*β*) = 0.8, respectively, resulting 26:26 samples in both groups were required. Accordingly, each of 29 highly-skilled and 30 less-skilled groups selected in this study justify unpaired t-test undertaken.

### Procedures

As illustrated in Fig 1, participants placed their hands shoulder width apart on the middle of the force platform with their feet placed on the floor (off the force platform) before performing the SAPH. Participants were instructed to hold the final handstand position for at least 5 seconds. Participants performed two SAPH trials and with better quality trial, as evaluated by the participant through self-reflection was analyzed. Measurements of ground reaction forces were carried out using a force platform (Kistler Inc., Type 9287C) at a sampling rate of 1 kHz. Simultaneously, images were captured from a distance of 10 meters using a video camera (Basler AG., Type A602fc-2) with settings of 100 fps and a shutter speed of 1/1000 s. The camera was positioned on the left side of the subjects, perpendicular to the sagittal plane of movement. Force platform and image data were synchronized using the A/D conversion video integration system TRIAS (DKH Inc., Japan), and entered into a personal computer.

#### Data processing

The x-y coordinates for each landmark as well as force platform reference points were digitized on every frame of video film from toe-off to final handstand position of SAPH. Respective positions of anatomical landmark attachments were the vertex of the head, acromion process, twelfth thoracic vertebra, great trochanter, lateral epicondyle of femur, lateral malleolus, acropodion, lateral epicondyle of the humerus, ulnar styloid process, and head of the fifth metacarpal bone. Image calibration was carried out using 2.5 m string weights drop method with reflective marks 0.3 m apart on the center of the force plate and 0.6 m repeat position in both left and right from the center of the force plate. Coordinate transformation using the 2-dimensional direct linear transformation method (DLT) [23] was carried out using the calibration images. The position of each coordinate marker data was obtained using a coordinate digitizing system Frame-DIAS-V (DKH Inc., Japan). Raw coordinate data were smoothed using a fourth order, zero-lag Butterworth low-pass digital filter (cut-off frequency of 5 Hz) according to residual analysis [24]. Centre of mass (COM) of the subjects was calculated using Dempstar’s body segment parameters equations [25]. The center of pressure (COP) was smoothed using a fourth order, zero-lag Butterworth low-pass digital filter as cut-off frequency of 6 Hz in accordance with residual analysis. Both video cameras and force platform coordinates were established by setting the ulnar styloid process as “*the original point*”, with the anterior (the back when inverse) as plus (+x), and the posterior (stomach when inverse) as minus (-x) (Fig 1). The SAPH section selected for analysis was from the toe-off to full handstand, involving three time-position phases: ① toe-off, ② L-position (leg horizontal position), ③ handstand (Fig 1).

In a previous study of the handstand [9], fluctuations in COP and COM were considered as indicators of balance ability. In light of these data, fluctuations in COP during SAPH were evaluated using root mean square of the COP (RMSCOP) in the anterior-posterior direction from toe-off to the attainment of the final handstand position.

#### Inverse dynamics solution

**Fig 2:**
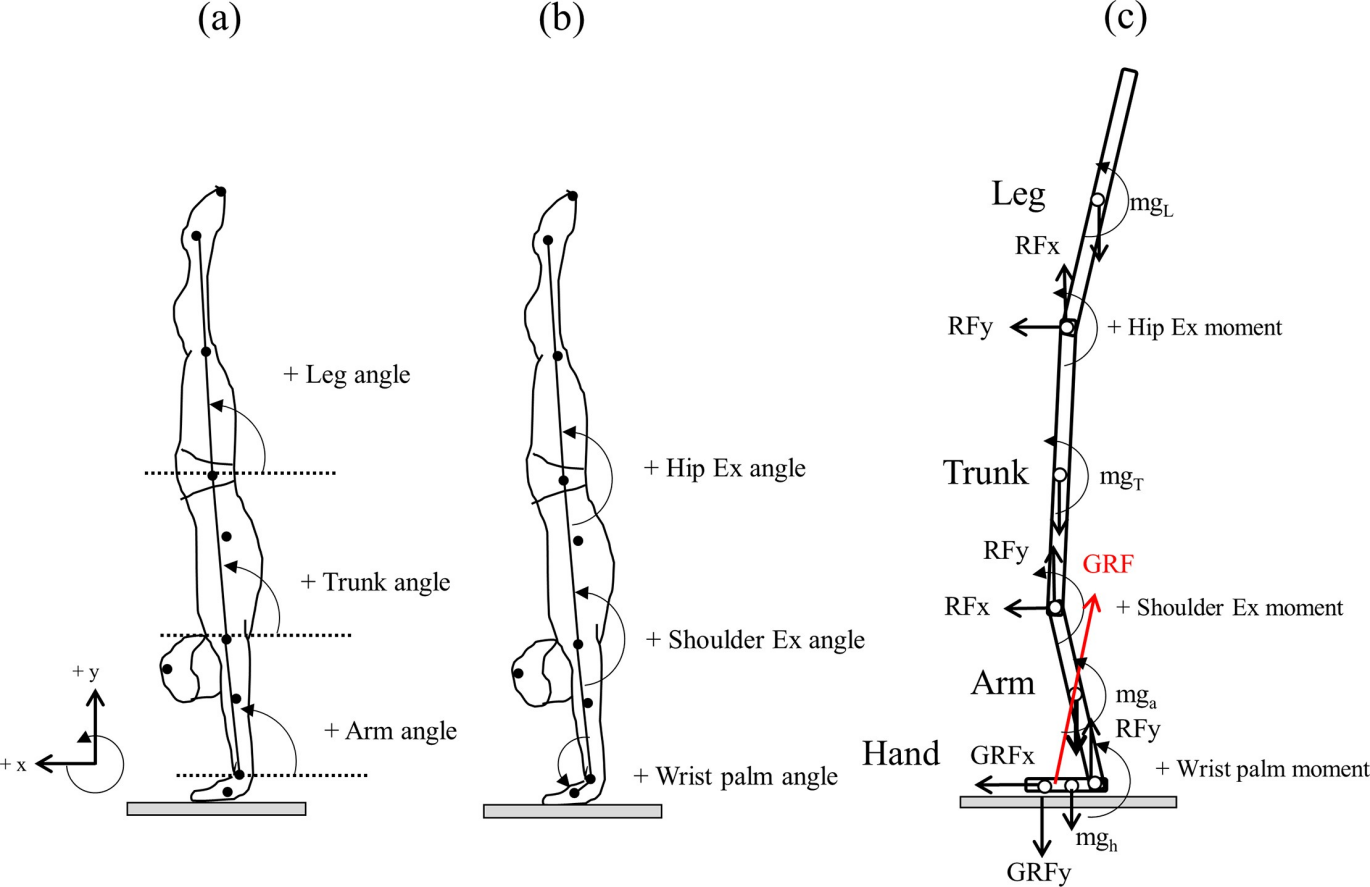
Definitions of kinematics and joints moment for inverse solution inputs in four segment model. Segments angles (a), joints angles (b) and joint reaction forces as well as moments around segment mass (c). Counterclockwise directions in segment angles and extensors in joint angles and moments assumes as plus sign, respectively.

Joint moments were calculated by means of two-dimensional inverse dynamics solution using the four-segment model (hands, arms, trunk and lower limbs) reported previously [10] (Fig 2). Body segment inertial properties were calculated using the methods as proposed by Dempstar [25]. Segment angles were referenced to the right horizontal and the body segment, where all movement was in a counter-clockwise direction designated as positive (+). Joint angles were designated as angles of the wrist joint (angle formed between hand and arm), shoulder joint (angle formed between arm and trunk), and hip joint (angle formed between trunk and leg), where the extensor direction designated as plus (+) (Fig 2). Kinematic and kinetic data were normalized as a time course of 100% from toe-off to upright handstand using a cubic spline.

### Statistical analysis

All measurable variables were expressed as average values and standard deviations. In order to compare the kinematic and kinetic data at toe-off, leg horizontal and handstand positions, Bonferroni adjustment method was introduced after significant differences were observed in one-way repeated ANOVA. The effect size(*η*^*2*^) was evaluated as 0.01 to 0.06 being small, 0.06 to 0.14 being medium, and 0.14 and above being large. An unpaired t-test was used to compare the difference between highly-skilled and less-skilled in movement time and RMS COP data. Statistical analysis was carried out using SPSS statistics V22 (IBM) as statistical significance level of *p* <0.05. In addition to the difference in unpaired t-test, the Cohen’s d as effect size [26] was calculated using the mean and standard deviation. The Cohen’s d as effect size(*d*) was evaluated as 0.2 to 0.5 being small, 0.5 to 0.8 being medium, and 0.8 and above being large. Differences in the kinematic and kinetics data between highly-skilled and less-skilled at every 1% time point of the normalized time were determined using a two-sample t-test of statistical parametric mapping (SPM). SPM can tightly control for the occurrence of type I errors, and the critical threshold was calculated based on a random field theory [27]. SPM procedures are conceptually identical to univariate procedures (e.g. univariate independent t-test), and the only apparent difference is that SPM uses a different probability distribution such as the random field theory [28]. If any values of SPM exceeded the critical threshold, then each kinematic and kinetics value at this point was considered significantly different between the groups. Exact *p*-values were computed for each suprathreshold cluster using cluster size and random field theory distributions for SPM topology. SPM analyses were implemented using the open-source SPM code (www.spm1d.org) in Matlab (R2015b; MathWorks Inc, Natick, MA, USA). Significance was set at *p* < 0.05 for comparisons.

## Results

**Fig 3:**
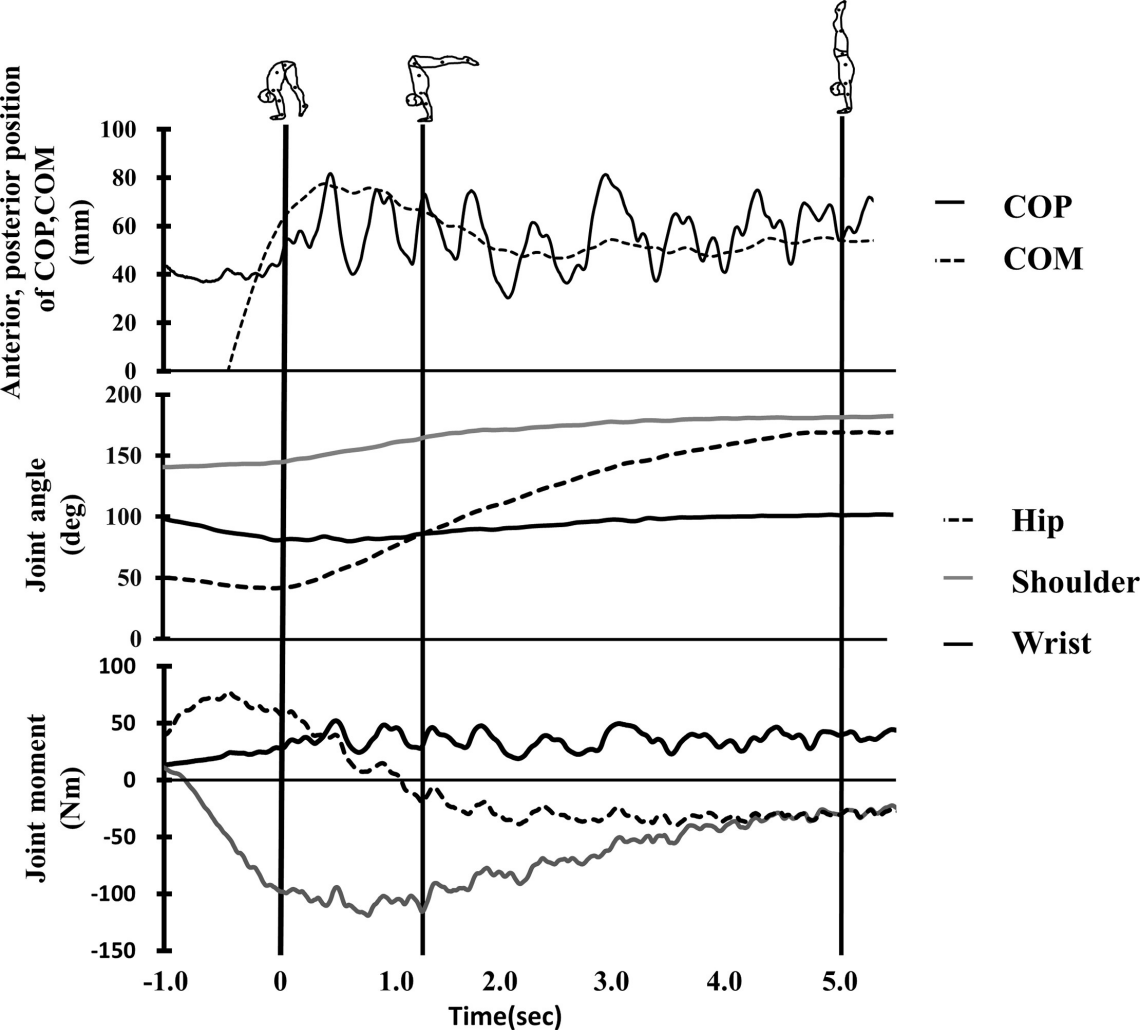
Typical examples of kinematics and kinetics during SAPH in a highly-skilled athlete. Origins of both COP and COM were set at styloid process of ulna’s y- coordinate. Vertical lines in the figure indicated respective three timings: Toe-off, Leg horizontal position and Handstand.

Fig 3 illustrates typical trajectory changes in COM and COP during the SAPH in a highly-skilled athlete. From toe-off to the L-position, COM was positioned posterior to COP. There were overlaps between the COM and COP trajectories from the L-position to the handstand. COM was positioned anterior to the COP when the handstand was completed.

**Fig 4:**
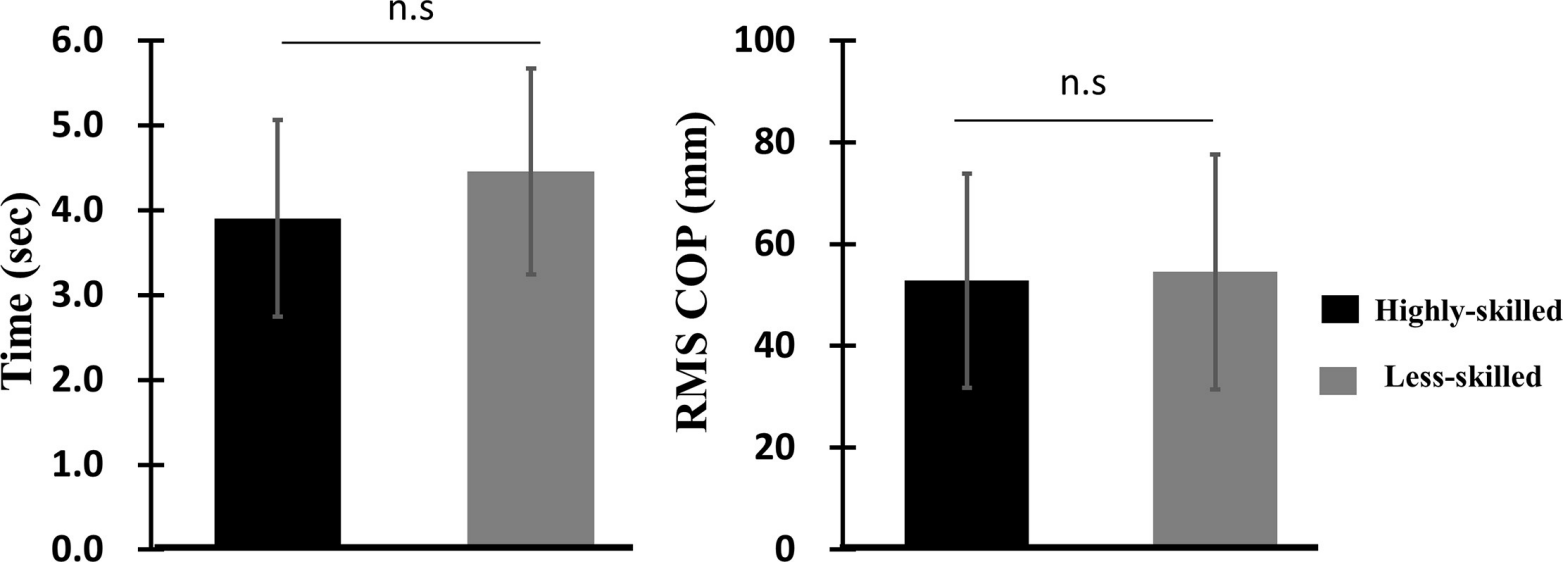
Comparisons of movement time and RMSs of COP fluctuations between highly- skilled and less-skilled during SAPH.

Hip joint angle had tendency to increase from toe-off to the handstand position (*F = 1687*, *p<0*.*001*, *η*^*2*^ *= 0*.*97*), whereas the wrist joint angle increased from toe-off to the handstand position (*F = 125*, *p<0*.*001*, *η*^*2*^ *= 0*.*68*, toe-off = L-position < Handstand). The shoulder joint angle had a tendency to increase from toe-off to L-position and thereafter maintained at the angle around 180 degrees (fully flexed position, *F = 675*, *p<0*.*001*, *η*^*2*^ *= 0*.*92*, toe-off < L-position < Handstand). Joint moments presented in Fig 3, indicated decrease positive wrist joint moment (*F* = *6*.*2*, *p<0*.*001*, *η*^*2*^ *= 0*.*1*, toe-off = L-position > Handstand) as well as negative shoulder joint moment(*F = 179*, *p<0*.*001*, *η*^*2*^ *= 0*.*76*, toe-off > L-position > Handstand). In contrast, hip joint moments changed from positive to negative during SAPH(*F = 438*, *p<0*.*001*, *η*^*2*^ *= 0*.*88*, toe-off > L-position > Handstand). Durations from toe-off to complete handstand in SAPH (*p* = 0.077, *d* = 0.47) as well as RMS COPs (*p* = 0.951, *d* = 0.08) were not different between highly- skilled and less-skilled (Fig 4).

**Fig 5:**
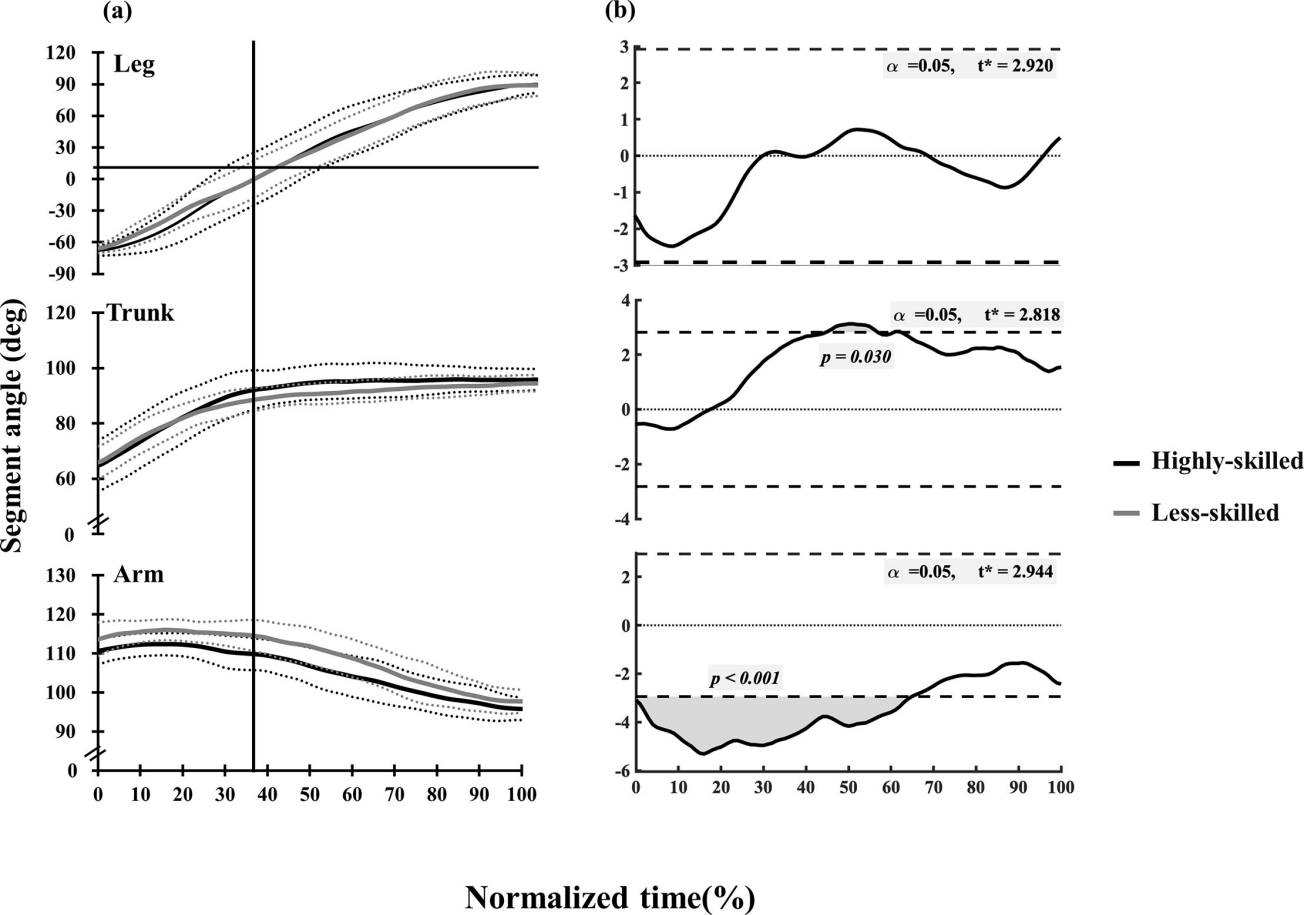
Changes in mean values of segment angles (a) and statistical parametric mapping (SPM) (b) between highly-skilled and less-skilled. Horizontal axis indicated normalized time of SAPH defined as 100% from Toe-off to Handstand. Dotted lines indicated mean ± one SD. Vertical line indicated the timing of Leg horizontal position. Associated SPM t-test results for differences between the curves in (b). The shaded area indicates suprathreshold clusters, which correspond to statistically significant differences between the curves at those nodes (percentage of normalized time). Asterisks indicated time zones when significant differences (*p* < 0.05) between highly-skilled and less-skilled were observed.

As for the arm segment angle, less-skilled performers displayed a significantly more forward position than those of highly-skilled performers from toe-off to the handstand (at 0–64%, *p* < 0.001, Fig 5). Conversely, there was a significantly different in the trunk angle between highly-skilled and less-skilled after the timing of the L-position; i.e. the highly-skilled group displayed a more vertical position when compared to less-skilled performers (at 45–57%, *p* = 0.03, Fig 5).

**Fig 6:**
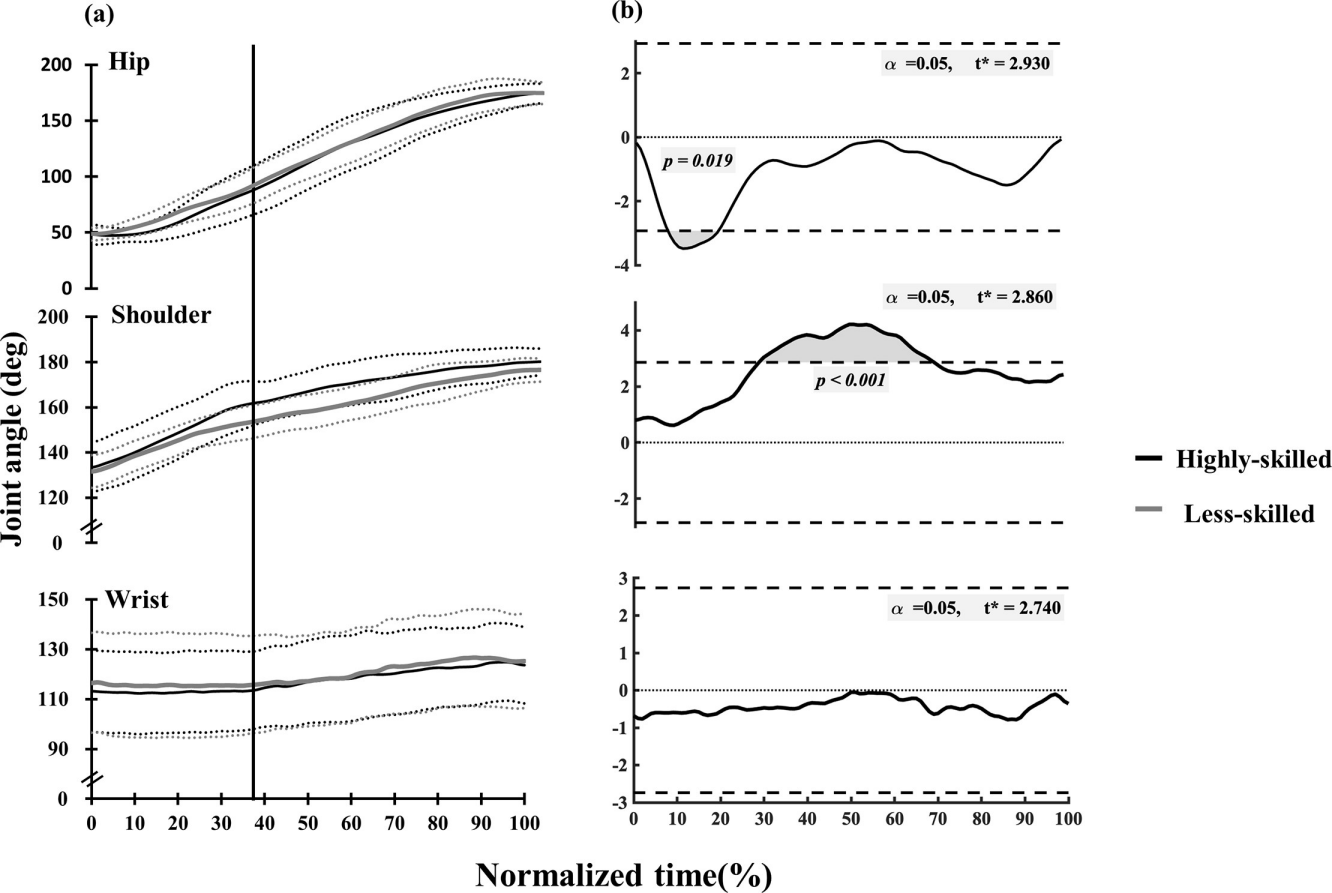
Changes in mean values of joint angles (a) and statistical parametric mapping (SPM) (b) between highly-skilled and less-skilled. Horizontal axis indicated normalized time of SAPH defined as 100% from Toe-off to Handstand. Dotted lines indicated mean ± one SD. Vertical line indicated the timing of Leg horizontal position. Associated SPM t-test results for differences between the curves in (A). The shaded area indicates suprathreshold clusters, which correspond to statistically significant differences between the curves at those nodes (percentage of normalized time).

There was no significant difference in wrist joint angle between the two groups from toe-off to the completed handstand. Highly-skilled performers showed a significantly larger shoulder flexion angle than the less-skilled gymnasts displayed from 29% to 69% normalization time (at 29–69%, *p* < 0.001, Fig 6). In contrast, the hip joint of the highly-skilled groups of performers displayed a significantly smaller flexion angle than less-skilled between 8% to19% of the normalization time (at 8–19%, *p* = 0.019, Fig 6). From these results, highly-skilled performed were characterized by a large shoulder flexion angle with a more flexed hip joint position, resulting in a more vertical position of the arm compared to the less-skilled performers.

**Fig 7:**
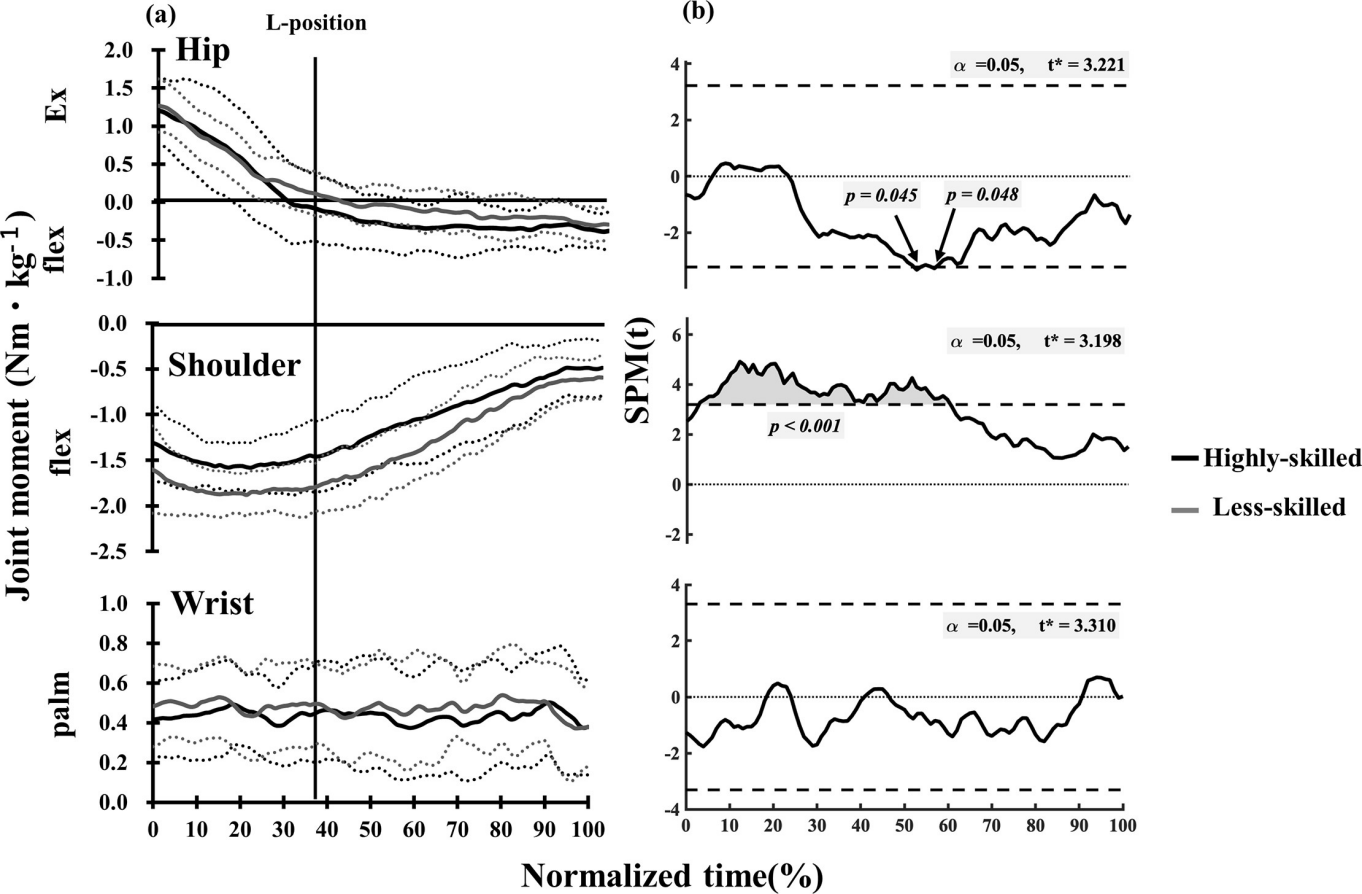
Changes in mean values of joint moments (a) and statistical parametric mapping (SPM) (b) between highly- skilled and less-skilled. Horizontal axis indicated normalized time of SAPH defined as 100% from Toe-off to Handstand. Dotted lines indicated mean ± one SD. Vertical line indicated the timing of Leg horizontal position. Associated SPM t-test results for differences between the curves in (b). The shaded area indicates suprathreshold clusters, which correspond to statistically significant differences between the curves at those nodes (percentage of normalized time).

Each joint moment pattern in groups, illustrated in Fig 7, represents the calculated output pattern time-normalized to 100% from toe-off to a completed handstand. Wrist joint moments were relatively constant around 0.4Nm・kg^-1^, showing no major differences between highly-skilled and less-skilled gymnasts. However, the less-skilled group produced higher magnitude shoulder flexor moments from 3% to 59% (*p*<0.01) normalized time when compared to the highly-skilled performer. Relatively constant hip moments were observed from toe-off to the L-position in both highly-skilled and less-skilled. After the beginning of the L-position at 52% (*p* = 0.045) and 56% (*p* = 0.048) normalized time, the highly- skilled group changed their hip joint moments from extensor to flexor, whereas the less-skilled group demonstrated zero or a small hip flexor moment. Hip flexor moments in the highly-skilled group were significantly larger compared to the less skilled performers.

## Discussion

This study attempted to clarify the characteristics of kinematic and joint moment profiles during SAPH in different skill levels in a group of Japanese top class and junior level male gymnasts. Successful SAPH techniques observed in highly-skilled performers were characterized as more acute pike position at toe-off as well as hip flexor moments at latter phase of SAPH.

Qualitative evaluations of SAPH in artistic gymnastics have been based on stability of dynamic balance as well as smoothness of motion without using extra muscle force. Results from this will provide insight into these qualitative evaluations by describing and analyzing the joint kinematics and moments profiles during SAPH as performed by highly-skilled and less-skilled male gymnasts. There is no significant difference between the groups in the results presented in Fig 4 as well as wrist joint angle and moment. We think the reason is to find in the good level of all gymnasts. If we use two levels of skilled gymnasts it is not easy to find differences, but the detected differences described below are more important than differences between poor (= unskilled, with problems to reach the handstand in the correct way) and good gymnasts.

Although the time of SAPH was short for highly-skilled performers, there was no statistically significant difference between the two groups (Fig 4). However, during competition, a long duration of SAPH would be disadvantageous to performance because the torque must be continuously maintained. Furthermore, although RMS in COP has been used as an indicator of balance in a previous report [6], it does not seem to be a good indicator of skill level in SAPH performance.

The magnitude and timing of each joint moment were expected to differ in this study between the highly-skilled and less-skilled performers. For example, from toe-off to L-position, less-skilled gymnasts produced significantly greater magnitudes of active shoulder joint flexion moments than the highly-skilled performers. This suggested that analysis of the differences in magnitude of shoulder moments might be an important variable indicative of SAPH skill level. In accordance with the handstand scoring rules, “Handstand elements must be achieved with completely straight arms and without any interruption of swing or obvious use of strength” [2, 6]. In order to deliver a performance of merit points, efficient production of the appropriate joints moments, at each joint at the right time, is requisite to successfully performing an element without an obvious use of strength. In this study, characteristics of the highly-skilled technique ability to maintain a small shoulder joint moment by keeping their arms and trunk as vertical as possible whereas in the less-skilled, greater shoulder flexion angle displacement as well as larger shoulder joint flexion moments. This may provide the evidence necessary to shown that the less-skilled gymnasts perform SAPH by utilizing extra force.

### Joint, segment angles and hip joint flexibility

Magnitude of the hip joint flexion angle in the fully-flexed hip joint flexed position, at the time of greatest hip joint flexion angle, influences the development of the shoulder joint moments [14], suggesting that flexibility of the hip joint might diminish the use of shoulder flexion moments, extra muscle forces. In this study, highly-skilled gymnasts demonstrated greater hip joint flexion than the less-skilled from 8% to 19% normalized time which is the time the hip joint reaches maximum flexion from toe-off to L-position. Large hip and shoulder joint flexion angles might be the results of trunk and arm angles being close to vertical in highly-skilled gymnasts (Fig 5). Hip joint flexibility might be needed to maintain large shoulder and hip joint flexion angle at toe-off. Previous research [14] suggests the importance of hip joint flexibility in the SAPH conducted on the parallel bars. Hip joint flexibility in hip instead of shoulder joint flexibility plays an important role from toe-off to L-position, resulting lower shoulder joint moment production in highly-skilled performers.

In this case, the main problem is the active flexibility not only passive flexibility which coaches stretch athletes’ shoulder. Active flexibility needs additional muscular strength i.e., to open the shoulder angle. However, due to the lack of published reports on joint flexibility, further research is need concerning hip and shoulder flexibility as related to joint moment production and performance of SAPH.

### Joint moments during SAPH

Prassas [14] reported that the magnitude of shoulder joint moments during the press with straight arm and bent body to handstand on the parallel bars were more than three times the body weight. The difference in starting position is thought to contribute to the difference in shoulder joint moments reported in this study(under 1.5Nm・kg^-1^ in Fig 7). It is conceivable that SAPH on the parallel bars compared to an SAPH in this study might create a more significant shoulder joint as the gymnast must raise one’s legs up from under the supporting bar to L-position.

A clear difference in hip joint moment production was detected with different skill levels from during the L-position to handstand phase (Fig 7). At the beginning of SAPH (toe-off-25% normalized time), both groups exhibited similar amounts of hip extension moments. However, in the highly-skilled, before the L-position at 29% normalized time, hip joint moments clearly changed from extension to flexion, whereas in the less-skilled, definite amount of flexion moments were not observed after L-position. Therefore, it can be inferred that the hip joint movement of highly-skilled acts as negative work, i.e., hip extensor muscles might behave lengthening (eccentric) action from before L-position to finish of SAPH. Even after the L-position, the trunk angle in the highly-skilled group was close to vertical, at around 90 degrees, whereas the less-skilled group presented with an angle of less than 90°. Therefore, it is conceivable that trunk angle differences from L-position-handstand movement are related to the hip joint moment differences between highly-skilled and less-skilled gymnasts.

Wrist joint moments were maintained at approximately 40 percent of the body weight, from the toe-off to handstand completion, with no statistically significant differences noted in either group. These values were similar to those of wrist joint moment examined in a still handstand [9]. In previous research of still handstands, wrist joint moment is responsible for anterior-posterior adjustment of the COM and moreover, highly-skilled gymnast exhibits smaller fluctuations in wrist joint moment [9, 12]. Rohleder, J. and Vogt, T. [6, 7] have also suggested the importance of wrist strategy for performance control in handstand in novice gymnasts. Wrist joint moment results from the current study failed to indicate skill difference as a definitive variable. However, highly-skilled performers maintained an equivalent level of stability both during the SAPH motion and in the still handstand. On the other hand, large variations in wrist joint moments were noted during SAPH in less-skilled. Possible causal factors for wrist joint moment differences could be poor posture due to either lack of flexibility or lack of inverted stability. The current study just quantified wrist joint moment during SAPH, illustrating that highly-skilled gymnasts maintained a constant value throughout, whereas less-skilled gymnasts showed large variations.

### Joint moments abilities and coordinated movement patterns in highly-skilled

**Fig 8:**
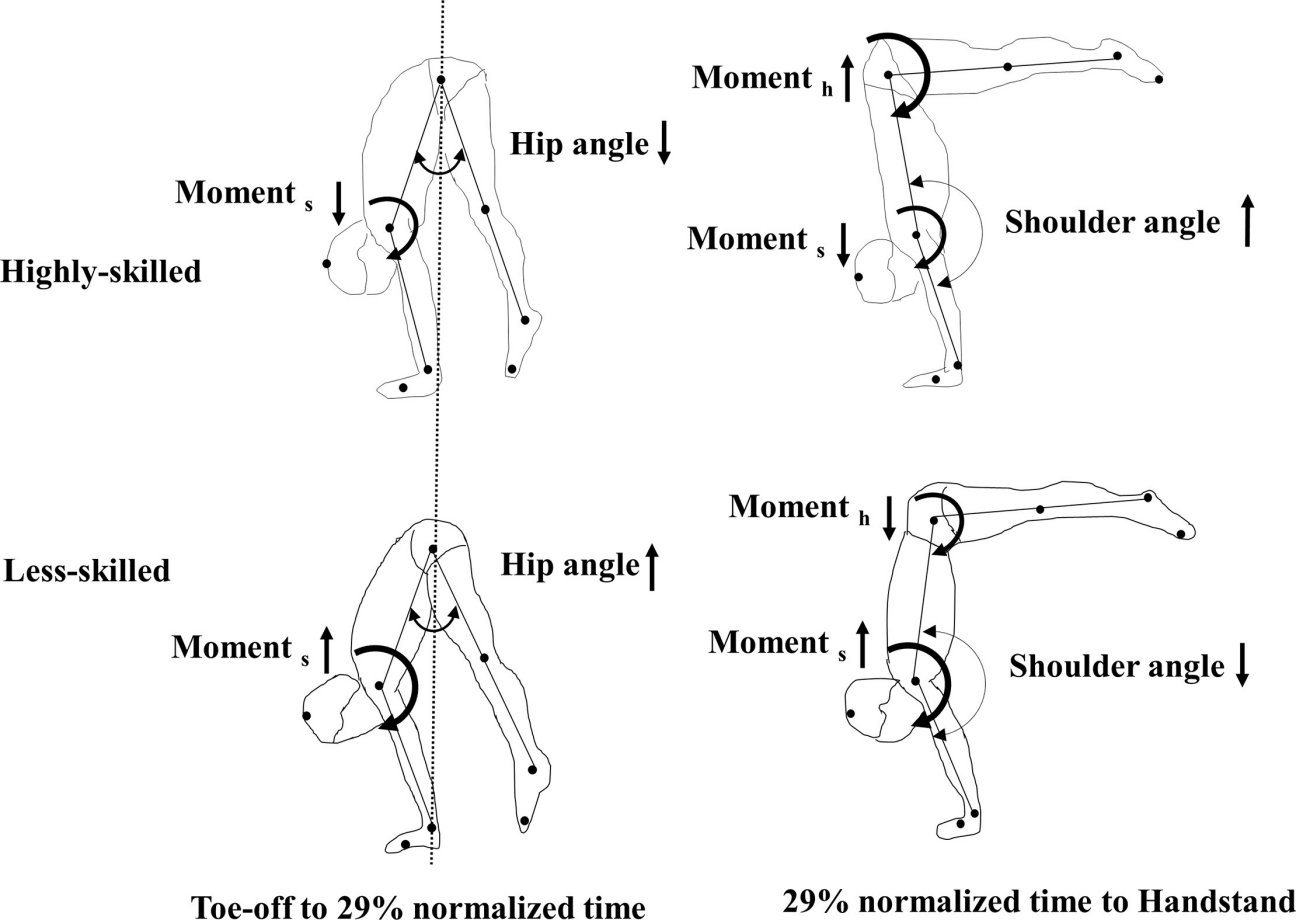
Schematic explanations showing the differences in kinematics and kinetics between highly-skilled and less-skilled during SAPH. Refer to text for more detailed interpretations.

Angle and joint moment changes for shoulder and hip joint, respectively, in highly-skilled and less-skilled as well as the moment productions at both toe-off and L-position are illustrated in Fig 8. The highly-skilled were characterized by a fully-flexed hip joint at toe-off phase and a large flexed angle in shoulder at L-position as well as a larger hip flexor moment production from L-position to handstand. Conversely, in the less-skilled, smaller hip flexed angle and large flexor moment in shoulder joint at toe-off phase as well as small flexor moment in hip joint at L-position were indicated. At toe-off position of SAPH, highly-skilled have greater flexibility in hip joint and can express a clear pike position by adjusted with less shoulder flexion moment and extend hip by exerting hip flexion moment (*hip strategy*). On the other hand, less-skilled start with more extend shoulder angle by exerting large shoulder flexion moment from L-position to handstand as well as extending hip joint with less hip flexion moment but large shoulder flexion moment (*shoulder strategy*).

Limitation of present study were lack of evidences for each joint moment production as well as flexibilities in shoulder and hip joints. Direct measurements of muscle activities such as EMG especially for shoulder and hip extensors/flexors might be needed in order to support differences in shoulder and hip moment productions observed between highly-skilled and less-skilled groups. On the other hand, direct measurement of joint flexibility is important factor for successful SAPH. New methods for the measurement and evaluation of flexibility might be developed for not only passive but also active (i.e. muscle strength to open shoulder angle) flexibilities in shoulder joint and for lumbo-pelvic plus hip joint flexibilities in hip joint which were observed at toe-off pike position in SAPH.

In summary, we investigated whether the magnitudes and timings of joint moment patterns at each joint influenced skill level during SAPH. The “highly-skilled” gymnasts were observed to reduce their shoulder joint flexion moments and transition from extension to flexion hip joint moment during the toe-off to the L-position phase. Generation of greater shoulder flexion moment after toe-off of SAPH was observed in the less-skilled performers. Therefore, smoothness of the movement might be able to change the hip moment from extension to flexion during leg rise phase of SAPH. A performer’s ability to coordinate joint moment patterns during training and subsequent competition relies on coaching regimens focused on training methods designed to have the gymnasts produce the required moment at the proper time during the movement. Evaluating a gymnast’s ability to coordinate moment patterns is imperative to the development of a “highly-skilled” performer. Description and analysis of the relationship between joint moments and moment profiles would enable the assessment of the performer’s ability to coordinate moment patterns, e.g. neural control, and their level of skill [22].

## Supporting information

S1 Data (XLSX)

## References

[1] Joshi HC, Ghai GD, Rajpoot YS. Kinematic analysis of close leg press handstand performing on different apparatuses in artistic gymnastics. International Journal of Yoga, Physiotherapy and Physical Education. 2017;2(4):7–10.

[2] 2017 Code of points, Men’s artistics gymnastics. FIG, Lausanne, (2017).

[3] 2001 Code of points, Men’s artistics gymnastics. FIG, Lausanne, (2001).

[4] 2006 Code of points, Men’s artistics gymnastics. FIG, Lausanne, (2006).

[5] Rohleder J, Vogt T. Efficacy of wrist strategy coaching on handstand performances in novices: inverting explicit and implicit learning of skill-related motor tasks. Science of Gymnastics Journal. 2019;11(2):209–222.

[6] Rohleder J, Vogt T. Performance control in handstands: challenging entrenched coaching strategies for young gymnasts. International Journal of Performance Analysis in Sport. 2018;18(1):17–31. 10.1080/24748668.2018.1440459.

[7] Rohleder J, Vogt T. Teaching novices the handstand: a practical approach of different sport-specific feedback concepts on movement learning. Science of Gymnastics Journal. 2018;10(1):29–42. 3a.

[8] Uzunov V. Developing the Straddle Sit Press to Handstand. Gym Coach. 2012;5:1–5.

[9] Kerwin DG, Trewartha G. Strategies for maintaining a handstand in the anterior-posterior direction. Medicine and science in sports and exercise. 2001;33(7):1182–8. Epub 2001/07/11. doi: 10.1097/00005768-200107000-00016 . 11445766

[10] Yeadon M, Trewartha G. Control strategy for a hand balance. Motor control. 2003;7(4):411–30. Epub 2004/03/05. . 14999137

[11] Slobounov S, Newell, KM. Postural Dynamics in Upright and Inverted Stances. Journal of applied biomechanics. 1996;12(2):185–96.

[12] Blenkinsop GM, Pain MTG, Hiley MJ. Balance control strategies during perturbed and unperturbed balance in standing and handstand. Royal Society Open Science. 2017;4(7):161018. Epub 2017/08/10. doi: 10.1098/rsos.161018 ; PubMed Central PMCID: PMC5541526. 28791131 PMC5541526

[13] Uzunov V. The handstand: A four stage training model. Gym Coach. 2008;2:52–9.

[14] Prassas S. A biomechanical analysis of the press handstand on the parallel bars utilizing inverse dynamics techniques.: Ph.D. Thesis, University of maeryland; 1985.

[15] Prassas S, Kelley DL., Pike NL. Shoulder joint torques and the straight arm/flexed hips press handstand on the parallel bars. International Symposium on Biomechanics in Sports 1986.

[16] Prassas S. Biomechanical modle of the press handstand in gymnastics. International Journal of sports biomechanics. 1988;4(4):326–41.

[17] Faria I. Parallel bars. In: RG B, editor. Sports technique. Northn Plam Beach, Florida: The Athletic institute; 1977. p. 35–7.

[18] Fukushima S, Russell W. Men’s gymnastics. London and Boston: Faber & Faber; 1980. p. 151–3.

[19] Brown JR., DB. W. Teaching and coaching gymnastics for men and women. New York: John Wiley & Sons; 1980. p. 331–2.

[20] Bawa G. Fundamentals of Men’s Gymnastics. New Dehli: Friends Publications; 1994.

[21] Özgören NS, Aritan S, editors. A Feedback Controller design for a Biomechanical Model of the Press Handstand in Gymnastics. 34 International Conference of Biomechanics in Sport; 2016; Tsukuba Japan2016.

[22] Kochanowicz A, Niespodzinski B, Mieszkowski J, Marina M, Kochanowicz K, Zasada M. Changes in the Muscle Activity of Gymnasts During a Handstand on Various Apparatus. J Strength Cond Res. 2019;33(6):1609–18. Epub 2017/07/13. doi: 10.1519/JSC.0000000000002124 . 28700510

[23] Abdel-Aziz YI, Karara HM. Direct linear transformation from comparator coordinates into object space coordinates in close-range photogrammetry. Proceedings of the symposium on Close-range photogrammetry. 1971:1–18. 10.14358/PERS.81.2.103.

[24] Winter DA. Biomechanics and motor control of human movement. 4th.ed. New York: John Wiley & Sons; 2009.

[25] Dempster WT. Space requirements of the seated operator: geometrical, kinematic, and mechanical aspects of the body with special reference to the limbs. Ohio, Wright Air Development Center: Wright-Patterson Air Force Base,; 1955. p. 55–159.

[26] Cohen J. Statistical power analysis for the behavioral sciences (2nd ed.). New York: Lawrence Erlbaum; 1998.

[27] Adler RJ, Taylor JE. Random fieldsand geometry. New York: Springer-Verlag 2007.

[28] Pataky TC, Robinson MA, Vanrenterghem J. Vector field statistical analysis of kinematic and force trajectories. Journal of Biomechanics, 46(14), 2394–2401 2013. doi: 10.1016/j.jbiomech.2013.07.031 23948374

